# Regulated and Non-Regulated Mycotoxin Detection in Cereal Matrices Using an Ultra-High-Performance Liquid Chromatography High-Resolution Mass Spectrometry (UHPLC-HRMS) Method

**DOI:** 10.3390/toxins13110783

**Published:** 2021-11-05

**Authors:** Aristeidis S. Tsagkaris, Nela Prusova, Zbynek Dzuman, Jana Pulkrabova, Jana Hajslova

**Affiliations:** Department of Food Analysis and Nutrition, Faculty of Food and Biochemical Technology, University of Chemistry and Technology, 16628 Prague, Czech Republic; Nela.Prusova@vscht.cz (N.P.); pulkrabj@vscht.cz (J.P.); hajslovj@vscht.cz (J.H.)

**Keywords:** mycotoxins, ergot alkaloids, ultra-high-performance liquid chromatography, high-resolution mass spectrometry, cereal

## Abstract

Cereals represent a widely consumed food commodity that might be contaminated by mycotoxins, resulting not only in potential consumer health risks upon dietary exposure but also significant financial losses due to contaminated batch disposal. Thus, continuous improvement of the performance characteristics of methods to enable an effective monitoring of such contaminants in food supply is highly needed. In this study, an ultra-high-performance liquid chromatography coupled to a hybrid quadrupole orbitrap mass analyzer (UHPLC-q-Orbitrap MS) method was optimized and validated in wheat, maize and rye flour matrices. Nineteen analytes were monitored, including both regulated mycotoxins, e.g., ochratoxin A (OTA) or deoxynivalenol (DON), and non-regulated mycotoxins, such as ergot alkaloids (EAs), which are analytes that are expected to be regulated soon in the EU. Low limits of quantification (LOQ) at the part per trillion level were achieved as well as wide linear ranges (four orders of magnitude) and recovery rates within the 68–104% range. Overall, the developed method attained fit-for-purpose results and it highlights the applicability of high-resolution mass spectrometry (HRMS) detection in mycotoxin food analysis.

## 1. Introduction

Cereals represent a food commodity with huge impact on human and livestock diet, providing a significant amount of protein globally [1]; indeed, it is expected that their production will be expanded up to 13% till 2027 [2]. Nevertheless, cereal matrices (in combination with environmental conditions) provide an excellent substrate for fungal growth, which, in turn, can result in contamination by toxic secondary fungal metabolites, the so-called mycotoxins. Unfortunately, mycotoxin-contaminated foodstuffs are commonly monitored in the food chain, impacting both consumer health, such as the recent intoxication cases due to deoxynivalenol (DON) in China [3], and jeopardizing market integrity, as in the case of the aflatoxin M1 scandal in some Balkan states [4]. Therefore, the development of analytical methods for accurate and specific mycotoxin detection in cereals is very important.

A large number of analytical methods for mycotoxin determination have been developed, with immunoassays and chromatographic analysis being the most common analytical choices [5]. In the first case, immunoassays are based on antibody recognition of a selected mycotoxin [6] and represent an affordable and simple approach that can be applied even at the point-of-need (PON) [7]. Nevertheless, most of the mycotoxin immunoassays are singleplex, meaning that only one analyte can be detected per run; they also face specificity problems due to cross reactivity with compounds structurally similar to the analyte and their results are commonly (semi)-quantitative [8]. Consequently, they are mostly preferred to deliver rapid results that need to be confirmed by instrumental analysis. In terms of chromatographic methods, liquid chromatography tandem mass spectrometry (LC-MS/MS) is the golden standard in mycotoxin analysis, providing excellent performance characteristics [9]. This approach is widely preferred in the regulatory control of such contaminants as it fulfills all the requirements of the available legislation, such as Decision 2002/657/EC on performance of analytical methods and Regulation EC 1881/2006 on mycotoxin maximum levels (MLs). However, a trend using high-resolution MS (HRMS) methods, such as time-of-flight (ToF) MS or hybrid quadrupole orbitrap MS (q-Orbitrap), has been noticed [10]. These MS analyzers, besides achieving satisfactory targeted analyte screening (fulfilling regulatory requirements), also permit analyte detection without extensive method tuning and retrospective data mining, features of utmost importance considering the occurrence of new or emerging mycotoxins (or some of their transformation products); i.e., analytes for which analytical standards are commonly not available [11].

**Figure 1:**
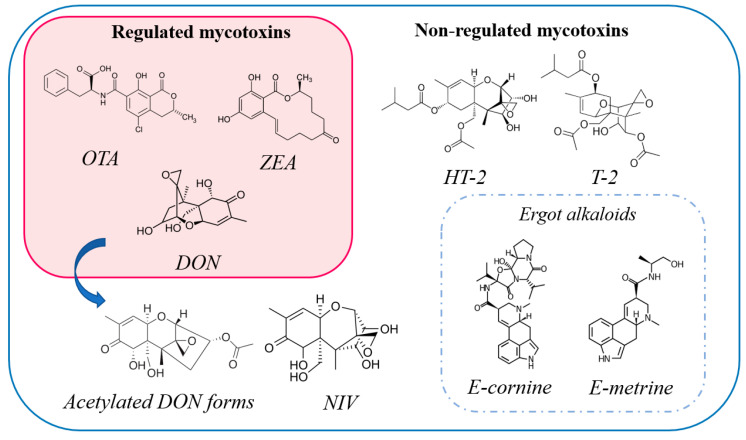
Chemical structures of the analytes investigated in this study.

In this study, an ultra-high-performance liquid chromatography coupled to a hybrid quadrupole orbitrap mass analyzer (UHPLC-q-Orbitrap MS) method was optimized and validated in wheat, maize and rye matrices. The analyte list contained 19 mycotoxins (Figure 1), namely, 3 regulated mycotoxins (ochratoxin A, deoxynivalenol and zearalenone) and 16 non-regulated mycotoxins, including 11 ergot alkaloids (EAs). In contrast to our recent study that focused on mycotoxin determination using ambient MS [12], in which the EA concentration was reported as a sum, in this case the EA epitopes can be effectively identified and quantified. In addition, all the detected mycotoxins are considered compounds with significant toxicity, resulting in potential health effects upon certain dietary exposure. In detail, ochratoxin A (OTA) is related to hepatotoxic, teratogenic and immunotoxic effects [13], and the European Food Safety Authority (EFSA) Panel on Contaminants in the Food Chain (CONTAM Panel) recently complied a risk assessment concluding that more exposure data are needed to better understand the in vivo impact of OTA to humans [14]. Regarding mycotoxins produced by *Fusarium* species, deoxynivalenol (DON) and nivalenol (NIV), belonging in the type-B trichothecenes, induce ribotoxic stress, including inhibition of protein, DNA and RNA synthesis [15]. Besides DON, also its acetylated metabolites, namely, 3- and 15-acetyldeoxynivalenol (3-ADON, 15-ADON), are analytes of high interest, as they can be absorbed more rapidly than DON and be converted to the parental form during digestion [16]. In terms of zearalenone (ZEA), it has shown strong estrogenic and anabolic effects [17] whilst the T-2 and HT-2 toxins, the most prevalent type-A trichothecenes, inhibit protein synthesis and target liver and spleen functions (mostly T-2 toxin) [18]. Last but not least, EAs produced by *Claviceps* species can cause ergotism, one of the oldest known human diseases caused by mycotoxins [19]. All in all, the described analyte toxic potential and their occurrence in the food chain (see Section 2) indicates the need to monitor these analytes and the present study provides an efficient and reliable analytical strategy to achieve it.

## 2. Results and Discussion

The development and validation of a fit-for-purpose method for the determination of 19 mycotoxins was achieved in the current study. Among them, three analytes were regulated, namely, DON, OTA and ZEA (Regulation EC 1881/2006), whilst only indicative levels for cereals and cereal products are available for the HT-2 and T-2 toxins (Recommendation 2013/165/EU). Importantly, although MLs were set for DON, OTA and ZEA, several exceedances were reported in the Rapid Alert System for Food and Feed (RASSF) EU portal (https://webgate.ec.europa.eu/rasff-window/screen/search, last accessed 11 October 2021) for all three analytes around Europe, including some in the Czech Republic. In terms of EAs, these are common rye contaminants, produced by *Claviceps purpurea,* but also other cereals can be contaminated by them, such as wheat [20]. Despite being non-regulated in the EU, the German Federal Institute for Risk Assessment (BfR) has issued “guidance levels” on EAs in cereal flours [21] and the Standing Committee on Plants, Animals, Food and Feed of the European Commission recently discussed (February 2021) the enforcement of MLs for ergot alkaloids (https://ec.europa.eu/food/system/files/2021-04/reg-com_toxic_20210226_sum.pdf, last accessed 11 October 2021). Furthermore, EFSA recently launched (February 2021) a call for data collection of chemical contaminants occurrence in the food chain, including ergot alkaloids (https://www.efsa.europa.eu/en/call/call-continuous-collection-chemical-contaminants-occurrence-data-0, last accessed 11 October 2021). Worthy to notice is that although LC-HRMS methods for mycotoxin analysis in cereals were earlier published (see Introduction), they either did not target all the ergot alkaloids considered for EU regulations [22,23] or their detectability was worse [24] in comparison to the presented study. In fact, excellent analytical performance was achieved for all the analytes (see Section 2.1) and the method trueness was further demonstrated by analyzing the proficiency testing (PT) samples, attaining successful results. In the last part of this paragraph (see Section 2.2), critical comparison towards already established LC-based methods is presented to highlight the merits and challenges of the proposed in-house method.

### 2.1. UHPLC-q-Orbitrap MS Method Optimization and Validation

**Figure 2:**
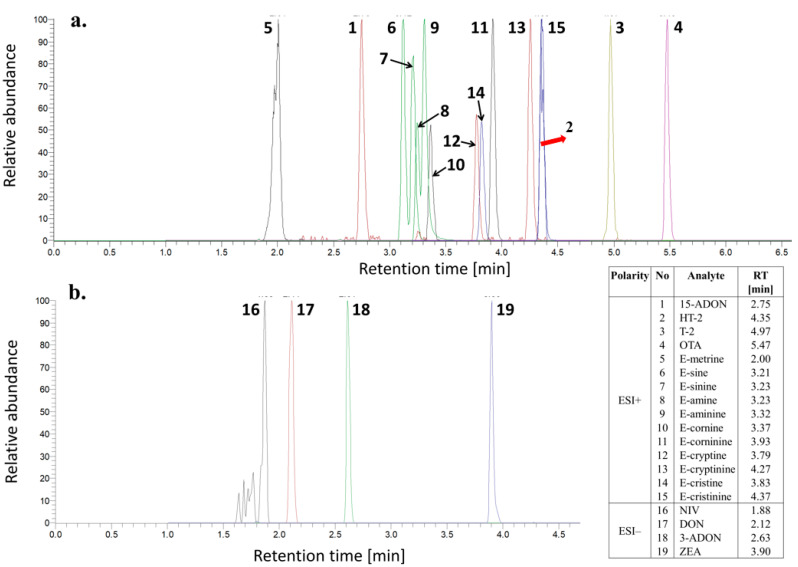
Extracted ion chromatograms (XICs) for the 19 analyzed mycotoxins in the wheat extract (concentration of each analyte 100 µg kg^−1^): (**a**) the ESI (+) ionization mode, and (**b**) the ESI (−) ionization mode.

One of our objectives was to develop a high-throughput method aiming to deliver a highly effective analytical tool intensifying mycotoxin testing. All 19 mycotoxins targeted in our study were eluted in less than 7 min in both polarity modes using an UHPLC-q-Orbitrap MS system. Mycotoxins were detected after fragmentation (parallel reaction monitoring, PRM mode) and normalized collision energies (NCEs) were optimized for each analyte in the range of NCE 10–100%, with a step of 10%. The optimal NCE was selected to provide the highest possible signal for at least two fragment ions (Table 1). Importantly, all analytes were confirmed following the criteria stated in the updated Directorate-General for Health and Food Safety (SANTE) guidelines (SANTE/12682/2019) on method validation for pesticide residues analysis in food and feed as there is no such guidelines for mycotoxin analysis [25]. The illustrative chromatogram of the wheat matrix-matched standard (Figure 2) depicts the efficient separation and sharp peak shape in most of the cases.

The multi-mycotoxin method was validated in wheat (Table 2), rye (Table 3) and maize flour (Table 4) matrices. Significantly, the attained LOQs were below the MLs set by the current EU legislation in cereal flours (Regulation EC 1881/2006). Satisfactory trueness expressed as recovery rate was achieved for all the analytes. In detail, the recoveries of the 19 analyzed mycotoxins at two spiking levels were in the range of 72–104% (L1) and 80–99% (L2) for wheat, 68–98% (L1) and 75–99% (L2) for maize and 69–102% (L1) and 75–104% (L2) for rye, respectively. Method repeatability expressed as RSD% fluctuated in the following range per case: 1–10% (L1) and 1–10% (L2) for wheat, 2–6% (L1) and 1–8% (L2) for maize and 1–9% (L1) and 1–7% (L2) for rye. In terms of method detectability, an extremely low LOQ was attained for OTA, ZEA and the 11 ergot alkaloids, specifically 0.5 μg kg^−1^, while in the case of trichothecenes, the LOQs were between 1 and 50 μg kg^−1^. Linear responses were acquired in all cases in the range LOQ–1000 µg kg^−1^, with a correlation coefficient (r^2^) of ˃0.999. The highest matrix effects % (MEs%) were noticed in rye extracts followed by maize and wheat extracts for all the studied analytes (Table 5). Specifically, considerable signal suppression was observed especially in the ESI (−), highlighting the need for utilizing matrix-matched calibration curves to compensate for the matrix effects. Such differences were expected as a generic sample preparation protocol was used and apparently the different cereals tested have different composition. Nevertheless, the already discussed satisfactory performance characteristics of the method indicate that such a generic sample preparation is fit for purpose. The possibility to use isotopically labeled internal standards (ISTDs) was not adopted since the cost of the method would have grown significantly, considering that this is a multi-mycotoxin method. Finally, to further demonstrate method trueness, we analyzed PT samples obtained within the FAPAS (FERA, York, UK) and RomerLabs (Romer Labs, Tulln, Austria) schemes. Seven different PT cereal samples were measured (Table 6), including 5 wheat and 2 maize flour samples, achieving acceptable results (z-score within the ±2 range in all cases).

### 2.2. Critical Comparison towards LC-Based Methods for Mycotoxin Detection

To compare the results attained by the in-house UHPLC-q-Orbitrap MS method towards already published studies, a critical discussion on important method characteristics for mycotoxin detection is presented. Given this context, it is needed to emphasize that the sample processing prior to instrumental analysis plays an important role. Focusing on studies published during the last four years, Quick, Easy, Cheap, Effective, Rugged, and Safe (QuEChERS) extraction has been commonly used, proving its wide acceptance in the field (see Table 7). Nevertheless, cereal matrices need further clean-up due to their high starch content and high amount of unsaponifiable lipophilic compounds, compounds that can decrease the analytical signal. In the reviewed literature, dispersive solid-phase extraction (dSPE) was applied as a clean-up step utilizing various sorbents. In detail, both conventional sorbents, such as primary secondary amine (PSA) [26] or zirconia-based (z-sep) [27], and newly introduced sorbents, such as MDN@Fe_3_O_4_ (a magnetic sorbent adsorbing hydrophobic and hydrophilic interferences) [28], were used, achieving great analytical performance in every case (Table 7). Alternatively, immunoaffinity column (IAC) clean-up was also used, acquiring analyte selective recognition due to the use of antibodies, for example in the case of DON [29]. However, it needs to be stated that commonly IAC significantly reduces the portfolio of analytes that can be detected (due to its selectivity) in a single run and thus such an approach is not preferable for multi-mycotoxin methods. In contrast to the aforementioned cases, in our study a freezing-out approach was used to eliminate the matrix co-extracted components such as lipids and other lipophilic compounds. In this way, a simple and cost-effective sample preparation protocol was applied.

Another important aspect impacting analytical performance is the method detector. Although studies using conventional detectors, for example fluorescence detector (FLD), are still being reported [29], MS detectors have been the most popular option, featuring unequivocal analyte identification and quantification. On the downside, MS detectors are costly, restricting their utilization in cases of limited resources, a fact that can pose a potential health threat to the population of such areas due to limited food testing (e.g., in African states [32,33]). The application of both low-resolution MS (LRMS) and high-resolution MS (HRMS) was reported for the determination of both regulated and emerging mycotoxins. In both cases, low LOQs, wide linear ranges and accurate results were acquired, characteristics of utmost importance in the food safety field. Despite using LRMS detectors, such as a triple quadrupole (QqQ), has been the golden standard; this preference is related to certain limitations. Considering that strong MEs (depending the food matrix) are commonly faced when using ESI, the lack of isotopically labelled mycotoxin ISTD pose a challenge in accurate quantification, especially in the case of ESI-QqQ [11]. Apparently, the use of matrix-matched calibration curves can partially solve this problem, but better results can be attained by using nano-LC systems or HRMS detection. Nano-LC permits high dilution of extracts, significantly decreasing the amount of ionizable matrix components; for example, a dilution factor of 40 was applied in a recent study to detect mycotoxins in various cereals [34]. In the case of HRMS, the accurate mass measurement (<5 ppm) and high resolution (>20,000 full width at half maximum (FWHM)) allow mycotoxin identification/quantification without (necessarily) the need for isotopically labelled ISTD. This is clearly demonstrated in our study, as excellent analytical performance was achieved, including LOQs at the part per trillion (ppt) level and wide linear range (four orders of magnitude), without using an isotopically labelled ISTD. In addition, HRMS enables retrospective data analysis, a feature that can be useful for conjugated mycotoxin detection. Conjugated mycotoxins are mycotoxin metabolites, usually connected to hydrophilic groups, formed during metabolism in order to reduce the parent compound toxicity [35]. However, such attached functional groups, e.g., glycosylic or sulfate moieties, are likely to be enzymatically cleaved during digestion upon consumption, resulting in additional dietary exposure to the precursor toxic mycotoxin [36]. Clearly, the use of HRMS methods for conjugated mycotoxin detection, for example, accurately screening such an analyte’s mass, is the only available option considering the lack of such analytical standards. In conclusion, the developed UHPLC-q-Orbitrap MS attained satisfactory results, comparable or even better than published studies, while its scope can be expanded to non-targeted screening.

## 3. Conclusions

The development and validation of an UHPLC-q-Orbitrap MS method for the detection of 19 mycotoxins in cereal matrices were presented. QuEChERS extract clean-up was performed by freezing-out, a simple and cost-efficient approach that was able to reduce lipid co-extracted matrix components. Importantly, the method provided rapid results (7 min in both polarity modes) and the attained LOQs were lower than the regulatory limits for all three regulated mycotoxins (OTA, DON and ZEA), indicating the method’s potential to be implemented in official food-control schemes. In terms of the non-regulated mycotoxins, excellent detectability was also achieved, a characteristic that can be useful in the effort to gather more occurrence data for non-regulated mycotoxins. Considering that there is discussion (in the EU) on setting MLs for some currently non-regulated mycotoxins, such as EAs, the current study acts proactively and delivers a method for their potential future regulatory control. In terms of ME, it was possible to quantify the analyte content accurately and precisely without employing isotopically labelled ISTD, due to the use of matrix-matched calibration curves. In conclusion, the presented study highlights the merits of HRMS in mycotoxin analysis and provides a comprehensive approach for the detection of high-interest analytes in cereals.

## 4. Materials and Methods

### 4.1. Chemicals

LC-MS grade methanol, acetonitrile, ammonium formate, ammonium acetate and formic acid were purchased from Sigma Aldrich (Taufkirchen, Germany). Deionized water (18.2 MΩ cm^−1^) was purified using a Milli-Q system (Millipore; Bedford, MA, USA). Analytical standards of mycotoxins DON, 3-ADON, NIV, 15-ADON, T-2, HT-2 and ZEA were purchased from Merck (Prague, Czech Republic, purity in the range 98.0–100.0%). EAs namely ergometrine (E-metrine), ergosine (E-sine), ergosinine (E-sinine), ergotamine (E-amine), ergotaminine (E-aminine), ergocornine (E-cornine), ergocorninine (E-corninine), ergocryptine (E-cryptine), ergocryptinine (E-cryptinine), ergocristine (E-cristine), ergocristinine (E-cristinine) were obtained by Romer Labs (Tulln, Austria, purity in the range 95.6–100.0%). The aforementioned standards were used to prepare a composite stock solution (5 µg mL^−1^ in acetonitrile), which was kept in a freezer (–20 °C).

### 4.2. Cereal Flour Samples

Wheat, rye and maize flour samples were bought from supermarkets and outdoor markets around Prague. The absence of mycotoxins in the purchased matrices was confirmed using the conditions described in [37] prior to method development and validation. To externally evaluate the trueness of the UHPLC-q-Orbitrap MS method, samples from the following PT schemes were analyzed: 17161, 22146, 22166 FAPAS wheat flour samples; 22134, 04384 maize flour samples (FERA, York, UK) and CSSMY018-M20161DZO, CSSMY020-M21161DZO wheat flour samples (Romer Labs, Tulln, Austria).

### 4.3. Sample Preparation

To extract the analytes, an optimized QuEChERS-based approach was used. Two grams of a cereal sample were weighed in a 50 mL centrifuge tube and 10 mL of acidified water (0.2% formic acid, v/v) were added, mixed and let to soak into the matrix for at least 30 min. For the extraction, 10 mL of acetonitrile were dispended, and samples were shaken for 30 min using a horizontal laboratory shaker (IKA Labortechnik, Staufen, Germany). To initiate phase separation, 4 g of magnesium sulphate (MgSO_4,_ Fluka, Buchs, Germany) and 1 g sodium chloride (NaCl, Penta, Chrudim, Czech Republic) were added and a tube was vigorously hand-shaken for 1 min. Phase separation was fully achieved by centrifugation at 10,000 revolutions per minute (rpm) (Rotina 380R, Hettich, Tuttlingen, Germany) for 5 min. In total, 5 mL of the supernatant were transferred into a 15 mL centrifuge tube and put into a freezer for 2 h to remove the co-extracted matrix components, such as lipids. Finally, the cleaned-up extract top layer was moved into a vial and was ready to be injected into the chromatographic system.

### 4.4. Ultra-High-Performance Liquid Chromatography Coupled to A Hybrid Quadrupole Orbitrap Mass Analyzer

An ultra-high-performance liquid chromatograph UltiMateTM 3000 (Thermo Scientific; Waltham, MA, USA) equipped with analytical column Acquity UPLC^®^ HSS T3 (100 × 2.1 mm, 1.8 µm; Waters, Milford, MA, USA) was used. Chromatographic conditions were adopted from our previous publication [37] and slightly modified, as described. Briefly, the column was held at 40 °C and temperature of the autosampler was at 10 °C. The mobile phases consisted of 5 mM ammonium formate and 0.2% formic acid, both in the Milli-Q water (A) and methanol (B) in the positive electrospray ionization (ESI (+)) and 5 mM ammonium acetate in Milli-Q water (C) and methanol (D) in the negative electrospray ionization (ESI (-)). Importantly, a minimal sample volume was needed in both polarity modes; in detail, 2 µL of the sample were injected into the system. Regarding ESI (+), the gradient started with 10% of B at 0.3 mL min^−1^, followed by a linear change to 50% of B and finally set to 100% of B in 8 min. Before injecting the next sample, it was necessary to wash the column with 100% of B for 2 min and to recondition for 2 min applying the initial conditions. In terms of ESI (–), the gradient conditions were (i) 10% of D with a flow of 0.3 mL min^−1^; (ii) increase to 50% of D after 1 min; and (iii) setting 100% of D to complete the chromatographic run. After completing the run, the chromatographic column was cleaned-up with 100% of D for 2 min and reconditioned for 2 min with the initial mobile phase composition.

Detection of mycotoxins was carried out using a high-resolution tandem mass spectrometer Q-Exactive PlusTM (Thermo Scientific, Waltham, MA, USA) equipped with Orbitrap-quadrupole mass filters. An overview of the applied mass spectrometric settings based on our previous study [38] is summarized in Table 8.

The detection of ions was performed in PRM mode in both polarity modes. The exact masses of the target analyte fragments were calculated in SW Xcalibur 4.2 (Thermo Scientific, Waltham, MA, USA) together with retention times and NCEs. Regarding the detection conditions, the resolution was set at 17,500 full width at half maximum (FWHM) (mass range *m/z* 50–1000 *m/z*), the maximum inject time (maxIT) was 50 ms and the automatic gain control target (AGC target) was equal to 1 × 10^5^. Lastly, Xcalibur 4.2 software was utilized to control the instrument and evaluate the attained data.

### 4.5. UHPLC-q-Orbitrap MS Validation

The UHPLC-q-Orbitrap MS method performance characteristics were investigated for three cereal flour matrices. Wheat, rye and maize flour samples containing non-detectable concentrations of mycotoxins were used. Matrix-matched calibration standards in the range 0.1–200 ng mL^−1^ (corresponding to 0.5–1000 µg kg^−1^) were prepared by evaporation of a composite analytical standard (at 5 µg mL^−1^) using a gentle nitrogen steam. Then, a blank matrix extract prepared according to the procedure described in the Section 4.3 was used for analyte reconstitution. Solvent standards in acetonitrile were prepared in the same concentration range to express the degree of MEs. The following formula was used to calculate the ME%:

ME% = [1 − (Peak area in the matrix-matched standard)/(Peak area in the standard)] × 100.

For the determination of trueness and repeatability, spiking was conducted in two levels, 250 µg kg^−1^ (level 1, L1) and 25 µg kg^−1^ (level 2, L2), both in six replicates. Trueness expressed as the recovery rate (R%) was calculated using the formula:

R% = (peak area of spiked sample/peak area of matrix-matched standard) × 100.

Repeatability was expressed as relative standard deviation % (RSD%) of these six replicates. Limits of quantification (LOQ) were determined as the lowest calibration points for a peak constructed at least from four points (no noise due to the high mass resolving power). The needed volume of composite stock solution (at 5 µg mL^−1^) was pipetted to 2 g of a blank sample (in a 50 mL centrifuge tube). Then, samples were vigorously hand shaken, left for 2 h to permit solvent evaporation and further processed, as described in Section 4.3.

## Figures and Tables

**Table 1:** Exact masses of the precursor and product ions of the targeted mycotoxins, as well as retention times and NCE.

Analyte	Retention Time (min)	Precursor ion		NCE (%)	Exact Masses of Fragments (*m/z*)	
		Type of Ion	Exact Mass (*m/z*)		1	2
15-ADON	2.75	[M + H]^+^	339.1704	10	321.1333	137.0597
HT-2	4.35	[M + NH_4_]^+^	442.2435	10	263.1278	215.1067
T-2	4.97	[M + NH_4_]^+^	484.2541	10	305.1384	245.1172
OTA	5.47	[M + H]^+^	404.0895	20	257.0211	239.0106
E-metrine	2.00	[M + H]^+^	326.1863	50	208.0757	223.1230
E-sine	3.13	[M + H]^+^	548.2867	30	223.1230	268.1444
E-sinine	3.21	[M + H]^+^	548.2867	30	223.1230	268.1444
E-amine	3.23	[M + H]^+^	582.2711	30	223.1230	297.1234
E-aminine	3.32	[M + H]^+^	582.2711	30	223.1230	208.0757
E-cornine	3.37	[M + H]^+^	562.3024	30	268.1444	223.1230
E-corninine	3.93	[M + H]^+^	562.3024	30	305.1285	223.1230
E-cryptine	3.79	[M + H]^+^	576.3180	30	268.1444	223.1230
E-cryptinine	4.27	[M + H]^+^	576.3180	30	223.1230	305.1285
E-cristine	3.83	[M + H]^+^	610.3024	30	223.1230	268.1444
E-cristinine	4.37	[M + H]^+^	610.3024	30	223.1230	305.1285
NIV	1.88	[M + CH_3_COO]^−^	371.1348	20	281.1031	311.1136
DON	2.12	[M + CH_3_COO]^−^	355.1398	10	265.1081	295.1187
3-ADON	2.63	[M + CH_3_COO]^−^	397.1504	10	307.1187	337.1293
ZEA	3.90	[M − H]^−^	317.1394	40	175.0401	131.0502

**Table 2:** UHPLC-q-Orbitrap-MS method validation data in the wheat flour matrix.

Analyte	Recovery ± RSD (%)		LOQ (µg kg^−1^)	Linear Range (µg kg^−1^)
	250 µg kg^−1^	25 µg kg^−1^		
NIV	72 ± 3	<LOQ	50.0	50–1000
DON	84 ± 3	80 ± 3	10.0	10.0–1000
3-ADON	86 ± 2	85 ± 5	5.0	5.0–1000
15-ADON	99 ± 10	85 ± 10	5.0	5.0–1000
HT-2	95 ± 3	103 ± 7	10.0	10.0–1000
T-2	89 ± 4	86 ± 7	1.0	1.0–1000
ZEA	91 ± 4	88 ± 6	0.5	0.5–1000
OTA	90 ± 2	88 ± 3	1.0	0.5–1000
E-metrine	79 ± 1	78 ± 2	0.5	0.5–1000
E-sine	81 ± 3	78 ± 5	0.5	0.5–1000
E-sinine	82 ± 3	85 ± 5	0.5	0.5–1000
E-amine	78 ± 4	85 ± 4	0.5	0.5–1000
E-aminine	81 ± 3	93 ± 2	0.5	0.5–1000
E-cornine	83 ± 3	80 ± 4	0.5	0.5–1000
E-corninine	88 ± 4	86 ± 7	0.5	0.5–1000
E-cryptine	94 ± 4	89 ± 5	0.5	0.5–1000
E-cryptinine	94 ± 2	91 ± 4	0.5	0.5–1000
E-cristine	90 ± 2	93 ± 2	0.5	0.5–1000
E-cristinine	88 ± 1	93 ± 3	0.5	0.5–1000

**Table 3:** UHPLC-q-Orbitrap-MS method validation data in the rye flour matrix.

Analyte	Recovery ± RSD (%)		LOQ (µg kg^−1^)	Linear Range (µg kg^−1^)
	250 µg kg^−1^	25 µg kg^−1^		
NIV	69 ± 2	-	50.0	50.0–1000
DON	89 ± 2	-	25.0	25.0–1000
3-ADON	88 ± 2	104 ± 3	5.0	5.0–1000
15-ADON	102 ± 9	92 ± 5	5.0	5.0–1000
HT-2	88 ± 3	82 ± 4	10.0	10.0–1000
T-2	104 ± 4	94 ± 2	1.0	1.0–1000
ZEA	92 ± 2	82 ± 2	0.5	0.5–1000
OTA	90 ± 2	90 ± 2	2.5	0.5–1000
E-metrine	80 ± 1	75 ± 1	0.5	0.5–1000
E-sine	82 ± 5	87 ± 3	0.5	0.5–1000
E-sinine	92 ± 3	99 ± 5	0.5	0.5–1000
E-amine	84 ± 5	99 ± 3	0.5	0.5–1000
E-aminine	90 ± 5	104 ± 4	0.5	0.5–1000
E-cornine	87 ± 2	82 ± 5	0.5	0.5–1000
E-corninine	92 ± 3	96 ± 1	0.5	0.5–1000
E-cryptine	86 ± 5	82 ± 4	0.5	0.5–1000
E-cryptinine	99 ± 3	90 ± 4	0.5	0.5–1000
E-cristine	93 ± 2	90 ± 5	0.5	0.5–1000
E-cristinine	95 ± 4	88 ± 1	0.5	0.5–1000

**Table 4:** UHPLC-q-Orbitrap-MS method validation data in the maize flour matrix.

Analyte	Recovery ± RSD (%)		LOQ (µg kg^−1^)	Linear Range (µg kg^−1^)
	250 µg kg^−1^	25 µg kg^−1^		
NIV	68 ± 4	-	50.0	50.0–1000
DON	81 ± 4	-	50.0	50.0–1000
3-ADON	86 ± 3	84 ± 7	2.5	2.5–1000
15-ADON	94 ± 3	-	25.0	25.0–1000
HT-2	81 ± 5	-	25.0	25.0–1000
T-2	95 ± 3	92 ± 5	2.5	2.5–1000
ZEA	92 ± 4	88± 7	0.5	0.5–1000
OTA	95 ± 4	80 ± 7	2.5	0.5–1000
E-metrine	96 ± 2	88 ± 1	0.5	0.5–1000
E-sine	81 ± 3	77 ± 5	0.5	0.5–1000
E-sinine	96 ± 3	83 ± 2	0.5	0.5–1000
E-amine	86 ± 6	83 ± 7	0.5	0.5–1000
E-aminine	93 ± 2	83 ± 1	0.5	0.5–1000
E-cornine	88 ± 3	82 ± 3	0.5	0.5–1000
E-corninine	89 ± 3	83 ± 4	0.5	0.5–1000
E-cryptine	87 ± 4	82 ± 6	0.5	0.5–1000
E-cryptinine	91 ± 2	92 ± 5	0.5	0.5–1000
E-cristine	95 ± 3	89 ± 4	0.5	0.5–1000
E-cristinine	94 ± 4	90 ± 8	0.5	0.5–1000

**Table 5:** Calculated matrix effects (ME%) for the 19 analytes in the corn, rye and maize flour extracts.

Analyte	ME%		
	Corn	Rye	Maize
NIV	37	43	39
DON	51	66	63
3-ADON	40	50	46
15-ADON	74	88	87
HT-2	58	82	81
T-2	67	109	108
ZEA	42	55	47
OTA	92	97	96
E-metrine	82	94	94
E-sine	57	90	66
E-sinine	74	132	93
E-amine	74	101	97
E-aminine	71	99	87
E-cornine	70	117	91
E-corninine	61	96	78
E-cryptine	74	120	99
E-cryptinine	64	99	88
E-cristine	81	104	95
E-cristinine	70	105	92

**Table 6:** Interlaboratory PT results attained by employing the in-house UHPLC-q-Orbitrap MS method.

Matrix	PT Sample	Analyte	Assigned Value (μg kg^−1^)	Measured Value (μg kg^−1^)	Z-Score
Wheat flour	FAPAS 22166	DON	708	789	0.7
		ZEA	76.2	100	1.4
		T-2	30.8	29	−0.3
		HT-2	20.8	20	−0.1
	FAPAS 17161	OTA	2.54	1.6	−1.7
	FAPAS 22146	DON	778	760	−0.1
		ZEA	87.6	94	0.2
		T-2	23.2	22	−0.2
		HT-2	32	36	0.6
	Romer Labs CSSMY018-M20161DZO	DON	854	1045	1.4
		ZEA	377	379	0
		OTA	21.9	22.8	0.2
	Romer Labs CSSMY020-M21161DZO	DON	2841	3267	1.1
		ZEA	179	177	0
		OTA	30.7	20.5	−1.5
maize flour	FAPAS 22134	NIV	135	116	−0.7
		DON	1320	1358	0.1
		3-ADON	60.6	63	0.3
		15-ADON	184	208	0.7
		T-2	309	247	−1.0
		HT-2	105	120	0.7
		ZEA	107	113	0.3
	FAPAS 04384	DON	859	1100	1.7
		ZEA	87.3	85.2	−0.1
		OTA	4.82	3.65	−1.1
		T-2	172	181	0.3
		HT-2	157	163	0.2

**Table 7:** Critical comparison to other LC-based methods.

Analytes	Matrix	Sample Preparation	Analytical Performance Characteristics				Isotopically Labelled ISTD	LC-Based Method	Ref
			Linear Range (r^2^ > 0.99)	R%	RSD%	LOQ (μg kg^−1^)			
8 emerging mycotoxins	cereal and cereal-based products	QuEChERS followed by dSPE (C18 and primary secondary amine)	linear responses for all the analytes	83–109%	<15%	0.01–7.19	no	UHPLC-QqQ-MS	[26]
12 ergot alkaloids	barley and wheat	acetonitrile-ammonium carbonate 5 mM (85–15, *v*/*v*) extraction, centrifugation, dSPE (C18/Z-sep + ), evaporation under nitrogen steam and reconstitution to methanol-water (1–1, *v*/*v*)	2–100 μg kg^−1^	84–104%	<11%	0.71–3.92 (barley) and 0.20–1.00 (wheat)	no	UHPLC-QqQ-MS	[27]
13 mycotoxins	feed	acetonitrile/water (80:20, *v*/*v*, 3% acetic acid) extraction in ultrasounds, magnetic sorbent clean-up, evaporation and reconstitution to methanol-water (1–1, *v*/*v*)	5–2500 μg kg^−1^	89–113%	<11%	0.2–40	no	UHPLC-QqQ-MS	[28]
DON and 3 DON conjugates	barley, wheat and maize	water extraction followed by n IAC clean-up	10–1000 μg kg^−1^	92–102%	<13%	10	no	HPLC-FLD	[29]
38 mycotoxins	cereal grains	QuEChERS-based with clean up. In case of HILIC analysis, the cleaned-up extract was evaporated under nitrogen steam and reconstituted to methanol-water (2–8, *v*/*v*)	0.05–2000 μg kg^−1^	61–120%	<15%	0.05–150	Deuterated ochratoxin d-4	UHPLC-QqQ-MS and HILIC-QqQ-MS	[30]
21 mycotoxins	gluten-free pasta	QuEChERS followed by extract dilution in deionized water (extract-water, 1–1, *v*/*v*)	0.25–1000 μg kg^−1^	71–125%	<11%	0.1–24	tentoxin-d3 ^13^ C_17_-tenuazonic acid, and ^13^ C_17_-aflatoxin B2	UHPLC-q-OrbitrapMS	[31]
19 mycotoxin and ergot alkaloids	wheat, rye, maize flour	QuEChERS followed by freezing out to remove co-extracted lipid components	0.5–1000 μg kg^−1^	68–104%	<10%	0.5–50	no	UHPLC-q-OrbitrapMS	This study

**Table 8:** Applied mass spectrometric conditions in this study.

Mass Spectrometric Conditions	
Sheath/auxiliary gas flow rate	45/10 arbitrary units
Capillary temperature	320 °C
Heater temperature	300 °C
Electrospray voltage	± 3.5 kV
S-lens value	55

## Data Availability

Data are available upon request; please contact the contributing authors.

## References

[1] Tao Y., Jia C., Jing J., Zhang J., Yu P., He M., Wu J., Chen L., Zhao E. (2021). Occurrence and dietary risk assessment of 37 pesticides in wheat fields in the suburbs of Beijing, China. Food Chem..

[2] (2018). Organisation for Economic Co-Operation Development-Food and Agricultural Organization (OECD-FAO).

[3] Ruan F., Chen J.G., Chen L., Lin X.T., Zhou Y., Zhu K.J., Guo Y.T., Tan A.J. (2020). Food Poisoning Caused by Deoxynivalenol at a School in Zhuhai, Guangdong, China, in 2019. Foodborne Pathog. Dis..

[4] Kerschke-Risch P. (2014). The aflatoxin-affair: The invisible victims of crime in the food-sector. Temida.

[5] Tsagkaris A.S., Nelis J.L.D., Ross G.M.S., Jafari S., Guercetti J., Kopper K., Zhao Y., Rafferty K., Salvador J.P., Migliorelli D. (2019). Critical assessment of recent trends related to screening and confirmatory analytical methods for selected food contaminants and allergens. TrAC Trends Anal. Chem..

[6] Nelis J.L.D., Tsagkaris A.S., Zhao Y., Lou-Franco J., Nolan P., Zhou H., Cao C., Rafferty K., Hajslova J., Campbell K. (2019). The End user Sensor Tree: An end-user friendly sensor database. Biosens. Bioelectron..

[7] Jafari S., Guercetti J., Geballa-Koukoula A., Tsagkaris A.S., Nelis J.L.D., Marco M.-P., Salvador J.-P., Gerssen A., Hajslova J., Elliott C. (2021). ASSURED Point-of-Need Food Safety Screening: A Critical Assessment of Portable Food Analyzers. Foods.

[8] Nolan P., Auer S., Spehar A., Elliott C.T., Campbell K. (2019). Current trends in rapid tests for mycotoxins. Food Addit. Contam. Part A.

[9] Weaver A.C., Adams N., Yiannikouris A. (2020). Invited Review: Use of technology to assess and monitor multimycotoxin and emerging mycotoxin challenges in feedstuffs. Appl. Anim. Sci..

[10] Tittlemier S.A., Brunkhorst J., Cramer B., DeRosa M.C., Lattanzio V.M.T., Malone R., Maragos C., Stranska M., Sumarah M.W. (2021). Developments in mycotoxin analysis: An update for 2019–2020. World Mycotoxin J..

[11] Vargas Medina D.A., Bassolli Borsatto J.V., Maciel E.V.S., Lanças F.M. (2021). Current role of modern chromatography and mass spectrometry in the analysis of mycotoxins in food. TrAC Trends Anal. Chem..

[12] Tsagkaris A.S., Hrbek V., Dzuman Z., Hajslova J. (2022). Critical comparison of direct analysis in real time orbitrap mass spectrometry (DART-Orbitrap MS) towards liquid chromatography mass spectrometry (LC-MS) for mycotoxin detection in cereal matrices. Food Control..

[13] Tao Y., Xie S., Xu F., Liu A., Wang Y., Chen D., Pan Y., Huang L., Peng D., Wang X. (2018). Ochratoxin A: Toxicity, oxidative stress and metabolism. Food Chem. Toxicol..

[14] Schrenk D., Bodin L., Chipman J.K., del Mazo J., Grasl-Kraupp B., Hogstrand C., Hoogenboom L., Leblanc J., Nebbia C.S., EFSA Panel on Contaminants in the Food Chain (CONTAM) (2020). Risk assessment of ochratoxin A in food. EFSA J..

[15] Bryła M., Ksieniewicz-Woźniak E., Waśkiewicz A., Szymczyk K., Jędrzejczak R. (2018). Natural Occurrence of Nivalenol, Deoxynivalenol, and Deoxynivalenol-3-Glucoside in Polish Winter Wheat. Toxins.

[16] Guo H., Ji J., Wang J., Sun X. (2020). Deoxynivalenol: Masked forms, fate during food processing, and potential biological remedies. Compr. Rev. Food Sci. Food Saf..

[17] Ropejko K., Twarużek M. (2021). Zearalenone and Its Metabolites—General Overview, Occurrence, and Toxicity. Toxins.

[18] Chen P., Xiang B., Shi H., Yu P., Song Y., Li S. (2020). Recent advances on type A trichothecenes in food and feed: Analysis, prevalence, toxicity, and decontamination techniques. Food Control..

[19] Agriopoulou S. (2021). Ergot Alkaloids Mycotoxins in Cereals and Cereal-Derived Food Products: Characteristics, Toxicity, Prevalence, and Control Strategies. Agronomy.

[20] Holderied I., Rychlik M., Elsinghorst P.W. (2019). Optimized analysis of ergot alkaloids in rye products by liquid chromatography-fluorescence detection applying lysergic acid diethylamide as an internal standard. Toxins.

[21] Oellig C., Melde T. (2016). Screening for total ergot alkaloids in rye flour by planar solid phase extraction–fluorescence detection and mass spectrometry. J. Chromatogr. A.

[22] León N., Pastor A., Yusà V. (2016). Target analysis and retrospective screening of veterinary drugs, ergot alkaloids, plant toxins and other undesirable substances in feed using liquid chromatography–high resolution mass spectrometry. Talanta.

[23] Liao C.-D., Wong J.W., Zhang K., Yang P., Wittenberg J.B., Trucksess M.W., Hayward D.G., Lee N.S., Chang J.S. (2015). Multi-mycotoxin Analysis of Finished Grain and Nut Products Using Ultrahigh-Performance Liquid Chromatography and Positive Electrospray Ionization–Quadrupole Orbital Ion Trap High-Resolution Mass Spectrometry. J. Agric. Food Chem..

[24] Bessaire T., Ernest M., Christinat N., Carrères B., Panchaud A., Badoud F. (2021). High resolution mass spectrometry workflow for the analysis of food contaminants: Application to plant toxins, mycotoxins and phytoestrogens in plant-based ingredients. Food Addit. Contam. Part A.

[25] The Directorate-General for Health and Food Safety (SANTE) (2019). SANTE/12682/2019. Analytical Quality Control and Method Validation Procedures for Pesticide Residues Analysis in Food and Feed. https://www.eurl-pesticides.eu/userfiles/file/EurlALL/AqcGuidance_SANTE_2019_12682.pdf.

[26] Kim D.-B., Song N.-E., Nam T.G., Lee S., Seo D., Yoo M. (2019). Occurrence of emerging mycotoxins in cereals and cereal-based products from the Korean market using LC-MS/MS. Food Addit. Contam. Part A.

[27] Carbonell-Rozas L., Mahdjoubi C.K., Arroyo-Manzanares N., García-Campaña A.M., Gámiz-Gracia L. (2021). Occurrence of Ergot Alkaloids in Barley and Wheat from Algeria. Toxins.

[28] Qian M., Yang H., Li Z., Liu Y., Wang J., Wu H., Ji X., Xu J. (2018). Detection of 13 mycotoxins in feed using modified QuEChERS with dispersive magnetic materials and UHPLC-MS/MS. J. Sep. Sci..

[29] Gonçalves C., Mischke C., Stroka J. (2020). Determination of deoxynivalenol and its major conjugates in cereals using an organic solvent-free extraction and IAC clean-up coupled in-line with HPLC-PCD-FLD. Food Addit. Contam. Part A.

[30] Rausch A.-K., Brockmeyer R., Schwerdtle T. (2020). Development and validation of a QuEChERS-based liquid chromatography tandem mass spectrometry multi-method for the determination of 38 native and modified mycotoxins in cereals. J. Agric. Food Chem..

[31] Tolosa J., Rodríguez-Carrasco Y., Graziani G., Gaspari A., Ferrer E., Mañes J., Ritieni A. (2021). Mycotoxin Occurrence and Risk Assessment in Gluten-Free Pasta through UHPLC-Q-Exactive Orbitrap MS. Toxins.

[32] Kemboi D.C., Ochieng P.E., Antonissen G., Croubels S., Scippo M.-L., Okoth S., Kangethe E.K., Faas J., Doupovec B., Lindahl J.F. (2020). Multi-Mycotoxin Occurrence in Dairy Cattle and Poultry Feeds and Feed Ingredients from Machakos Town, Kenya. Toxins.

[33] Olopade B.K., Oranusi S.U., Nwinyi O.C., Gbashi S., Njobeh P.B. (2021). Occurrences of Deoxynivalenol, Zearalenone and some of their masked forms in selected cereals from Southwest Nigeria. NFS J..

[34] Reinholds I., Jansons M., Fedorenko D., Pugajeva I., Zute S., Bartkiene E., Bartkevics V. (2021). Mycotoxins in cereals and pulses harvested in Latvia by nanoLC-Orbitrap MS. Food Addit. Contam. Part B.

[35] Berthiller F., Schuhmacher R., Adam G., Krska R. (2009). Formation, determination and significance of masked and other conjugated mycotoxins. Anal. Bioanal. Chem..

[36] Jai A.E., Zinedine A., Juan-García A., Mañes J., Etahiri S., Juan C. (2021). Occurrence of Free and Conjugated Mycotoxins in Aromatic and Medicinal Plants and Dietary Exposure Assessment in the Moroccan Population. Toxins.

[37] Dzuman Z., Zachariasova M., Lacina O., Veprikova Z., Slavikova P., Hajslova J. (2014). A rugged high-throughput analytical approach for the determination and quantification of multiple mycotoxins in complex feed matrices. Talanta.

[38] Dzuman Z., Zachariasova M., Veprikova Z., Godula M., Hajslova J. (2015). Multi-analyte high performance liquid chromatography coupled to high resolution tandem mass spectrometry method for control of pesticide residues, mycotoxins, and pyrrolizidine alkaloids. Anal. Chim. Acta.

